# The LINC complex component Sun4 plays a crucial role in sperm head formation and fertility

**DOI:** 10.1242/bio.015768

**Published:** 2015-11-30

**Authors:** Elisabeth Pasch, Jana Link, Carolin Beck, Stefanie Scheuerle, Manfred Alsheimer

**Affiliations:** Department of Cell and Developmental Biology, Biocenter, University of Würzburg, Würzburg D-97074, Germany

**Keywords:** LINC complex, Nuclear envelope, Sperm head formation, Spermiogenesis, SUN domain proteins

## Abstract

LINC complexes are evolutionarily conserved nuclear envelope bridges, physically connecting the nucleus to the peripheral cytoskeleton. They are pivotal for dynamic cellular and developmental processes, like nuclear migration, anchoring and positioning, meiotic chromosome movements and maintenance of cell polarity and nuclear shape. Active nuclear reshaping is a hallmark of mammalian sperm development and, by transducing cytoskeletal forces to the nuclear envelope, LINC complexes could be vital for sperm head formation as well. We here analyzed in detail the behavior and function of Sun4, a bona fide testis-specific LINC component. We demonstrate that Sun4 is solely expressed in spermatids and there localizes to the posterior nuclear envelope, likely interacting with Sun3/Nesprin1 LINC components. Our study revealed that Sun4 deficiency severely impacts the nucleocytoplasmic junction, leads to mislocalization of other LINC components and interferes with the formation of the microtubule manchette, which finally culminates in a globozoospermia-like phenotype. Together, our study provides direct evidence for a critical role of LINC complexes in mammalian sperm head formation and male fertility.

## Abstract (precis)

**Summary:** SUN-domain protein Sun4 is essential for sperm head formation. Sun4 deficiency interferes with the organization of the microtubule manchette and leads to a globozoospermia-like phenotype and infertility.

## INTRODUCTION

Spermatogenesis is the fundamental process by which undifferentiated primordial germ cells develop into mature spermatozoa. After several rounds of mitotic proliferation the spermatogonia enter meiosis and, by two rounds of meiotic divisions, produce haploid spermatids, which during spermiogenesis differentiate into fertilization-competent spermatozoa. Sperm formation includes assembly of sperm-specific structures like the flagellum and the acrosome, cellular and nuclear polarization, chromatin compaction and nuclear reshaping from initially round to elongated (Russell et al., 1991; Kierszenbaum et al., 2003; Kierszenbaum and Tres, 2004; Hermo et al., 2010a,b). Notably, failures affecting formation and regular shaping of the sperm head are identified as frequent cause for male infertility (Yan, 2009).

Nuclear restructuring during sperm head formation coincides with modulation of the nuclear envelope (NE), which in turn is presumed to be critical for transferring cytoskeletal forces, required for directed nuclear shaping (Göb et al., 2010; Kierszenbaum et al., 2011). One of the major characteristics is the redistribution and polarization of numerous NE components as determined for lamins B1 and B3, Lap2 and lamin B receptor, which all relocate to the posterior spermatid NE (Alsheimer et al., 1998; Mylonis et al., 2004; Schütz et al., 2005). In addition, two distinct LINC (linker of nucleoskeleton and cytoskeleton) complexes have previously been identified as further components of the spermatid NE, which also show polarization during sperm head formation: Sun3/Nesprin1 LINC complexes locate to the posterior NE, whereas Sun1η/Nesprin3 complexes are found at the opposite, anterior pole in late spermatids (Göb et al., 2010).

LINC complexes are found in all different kinds of cells. They were identified as NE-bridging assemblies connecting the nuclear content to the cytoskeleton. These bridges are formed by interaction of two evolutionarily conserved transmembrane protein families: the SUN and KASH domain proteins (Crisp et al., 2006; Stewart-Hutchinson et al., 2008; Razafsky and Hodzic, 2009; Starr and Fridolfsson, 2010). LINC complexes are crucial for many fundamental cellular processes like nuclear migration, anchoring and positioning. They are key mediators for force transmission across both nuclear membranes, which in particular is required for chromosome movement during meiosis, and they are central determinants for NE integrity, essential for maintaining general nuclear morphology and shape (Razafsky and Hodzic, 2009; Starr and Fridolfsson, 2010; Kracklauer et al., 2013).

SUN proteins, which share a common C-terminal motif, the SUN (Sad-1/UNC-84) domain, represent the inner nuclear membrane (INM) constituents of LINC complexes (Hagan and Yanagida, 1995; Malone et al., 1999). The mammalian genome contains five distinct genes coding for SUN proteins: *Sun1* and *Sun2* (Hodzic et al., 2004; Padmakumar et al., 2005), which are widely expressed in different cell types, and *Sun3*, *Sun4* (*Spag4*) and *Sun5* (*Spag4L*), which were described as testis-specific (Shao et al., 1999; Göb et al., 2010; Frohnert et al., 2011). Within the perinuclear space (PNS) SUN proteins interact via their SUN domain with the KASH (Klarsicht/ANC-1/Syne/homology) domain of KASH protein partners. These reside within the outer nuclear membrane (ONM) and connect to the cytoskeleton (Starr, 2009; Sosa et al., 2012). In mammals five different KASH proteins are known: Nesprin1 (Syne-1) and Nesprin2 (Syne-2), which both interact with actin and microtubule motor proteins (Zhang et al., 2001; Zhen et al., 2002; Cartwright and Karakesisoglou, 2014), Nesprin3, which is able to connect to plectin (Wilhelmsen et al., 2005; Ketema et al., 2007), the kinesin binding Nesprin4, (Roux et al., 2009), and the dynein-interacting meiosis-specific KASH5 (Morimoto et al., 2012).

Due to the general properties ascribed to LINC complexes they are prime candidates to play a vital role in sperm head formation. As previously shown, during the elongation process of spermatids Sun3/Nesprin1 and Sun1η/Nesprin3 LINC complexes locate at opposite poles. Therefore it was speculated that LINC complexes may operate as opposing nuclear anchors to transfer cytoskeletal forces, hence, enabling the gradual shaping and elongation of spermatid nuclei (Göb et al., 2010). In addition to Sun1/Sun1η and Sun3, yet another two SUN domain proteins have been found to be expressed during spermiogenesis: Sun5 (Spag4L) and Sun4 (Spag4). Like Sun1η, Sun5 (Spag4L) polarizes to the anterior pole of round and elongated spermatids (Frohnert et al., 2011; Kracklauer et al., 2013). The actual localization of Sun4 (Spag4) was not that clear; a previous study reported that in mammals at least a fraction of Sun4 localizes to the posterior NE of spermatids, just as shown for the *Drosophila* ortholog Spag4. However, in contrast to the *Drosophila* protein, a significant proportion of Sun4 may also be part of the outer dense fiber (ODF) (Shao et al., 1999; Kracklauer et al., 2010).

In the present study we started a detailed analysis of Sun4 to verify its actual expression pattern and localization, to determine its primary binding partners and to uncover its function in sperm formation and fertility. Our results show that Sun4 is a spermatid-specific protein, which selectively localizes to the posterior NE of round and elongating spermatids. We identified Sun4 as a mandatory partner of Sun3/Nesprin1 and found that Sun4 is crucial for correct assembly of the cytoplasmic microtubule manchette. By using a Sun4 knockout mouse line, we demonstrate its essential role in mammalian sperm development. Sun4 deficiency causes severe defects in sperm head formation, which lead to a globozoospermia-like phenotype and, thus, to complete male infertility.

## RESULTS

### Testis-specific Sun4 (Spag4) localizes to the posterior pole of spermatids

**Fig. 1.**
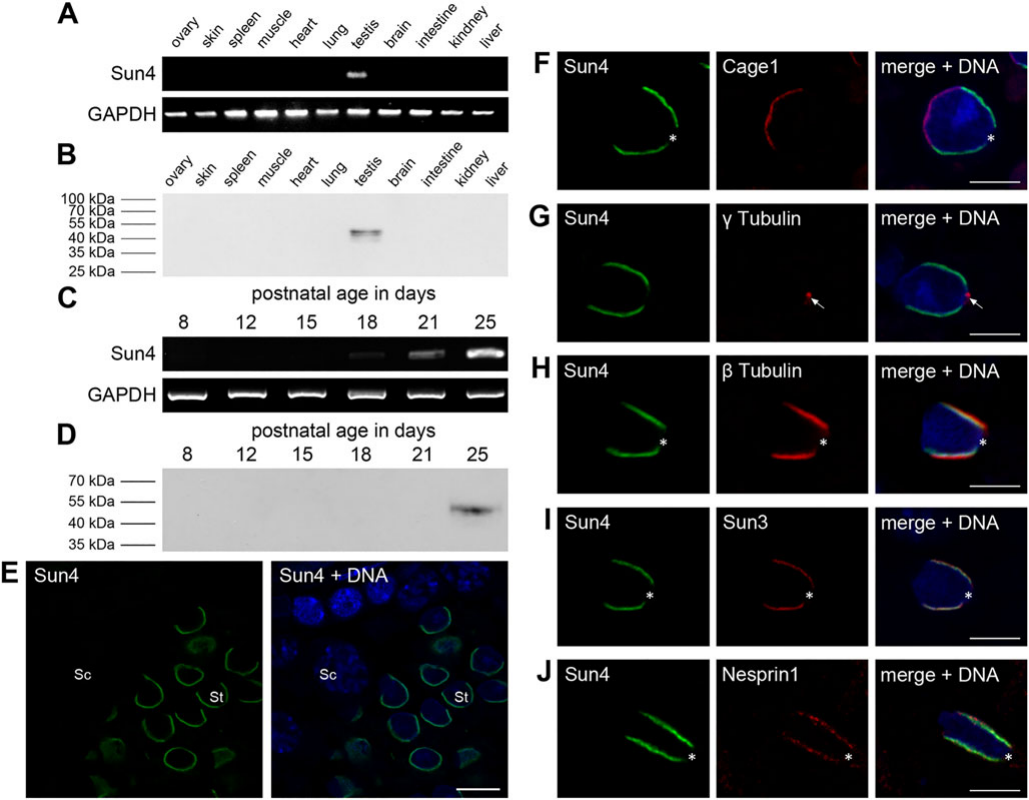
**Sun4 is spermiogenesis-specific and colocalizes with Sun3 and Nesprin1.** (A,B) Tissue-specific expression pattern of *Sun4* shown by (A) RT-PCR using *Sun4*-specific primers and (B) western blot using affinity purified anti-Sun4 antibodies. (C,D) Temporal expression pattern of Sun4 during mouse spermatogenesis analyzed by (C) RT-PCR using RNA of testicular cells from mice of different ages (day 8–25) and (D) immunoblot using equivalent amounts of cells of the same mice. (E) Immunolocalization of Sun4 on testis paraffin sections. Sc, spermatocyte; St, spermatid; Scale bar: 10 µm. (F-J) Co-immunofluorescence analysis of Sun4 in elongating spermatids, co-stained with (F) Cage1, (G) γ-tubulin, (H) β-tubulin, (I) Sun3 or (J) Nesprin1. Overlays are shown on the right; DNA is stained in blue. Asterisks mark the region of the implantation fossa, arrows indicate the basal body. Scale bars: 5 µm.

In a previous study, mammalian Sun4 (Spag4) was identified as a testis-specific protein, solely expressed in spermatids (Shao et al., 1999). To verify this finding, we initially performed RT-PCR on representative mouse tissues samples using *Sun4*-specific primers (Table S1). We could detect *Sun4* mRNA in the testis, but neither in any other somatic tissue nor in the ovary (Fig. 1A). To ascertain testis specificity, we performed western blot analysis using affinity-purified Sun4 antibodies, which recognized a protein with the expected molecular weight of 49 kDa in the testis tissue sample only (Fig. 1B).

To examine the *Sun4* expression profile in more detail we then performed RT-PCR on RNA from testis of 8- to 25- day old mice, covering the first wave of mouse spermatogenesis (Bellve et al., 1977; Malkov et al., 1998; Schütz et al., 2005). While in testes only containing premeiotic and meiotic stages, no *Sun4* transcript could be detected, a first faint signal appeared at day 18 postpartum, a time point when first postmeiotic stages are found. With spermiogenic progression at day 21-25, when spermatids numbers increase, *Sun4* signals became significantly stronger suggesting that *Sun4* is expressed in haploid stages only (Fig. 1C). This was confirmed by analyzing the expression profile of the Sun4 protein, which could only be detected in testicular suspensions at day 25, when spermatids are abundant within the testis (Fig. 1D).

To analyze Sun4 localization and behavior during spermatid differentiation, we next performed immunohistochemical experiments on paraffin sections of adult testis. While somatic cells, spermatogonia and spermatocytes were negative for Sun4 (Fig. 1E), a clear signal was observed at the NE of spermatids, confirming that Sun4 is only expressed during spermiogenesis (Fig. 1E). Notably, as described for other NE proteins in spermatids as well (Alsheimer et al., 1998; Mylonis et al., 2004; Schütz et al., 2005), Sun4 shows a clearly polarized localization. To study its precise distribution, we performed double-label immunofluorescence staining. Co-staining of Sun4 and acrosomal protein CAGE1, which localizes to the anterior pole of the developing sperm head (Alsheimer et al., 2005), revealed that Sun4 is only present at the posterior side of round and elongating spermatid nuclei (Fig. 1F). There it covered the more lateral regions, but was absent from the very posterior region of the implantation fossa as indicated by γ-tubulin staining of the adjacent basal body (Fig. 1G). It is worth noting that although we used three different antibodies directed against two distinct epitopes for Sun4 detection, we could never detect immunofluorescence staining of the axoneme (Fig. 1F-J, Fig. S1). This indicates that Sun4 is not actually present in the sperm tail, as was previously described by Shao et al., but rather is only part of the posterior nucleocytoplasmic junction as briefly mentioned in the previous study as well (Shao et al., 1999).

### Sun4 appears to be associated with the microtubule manchette and co-localizes with Sun3 and Nesprin1

A prominent structure of the developing spermatid is the manchette, which covers the posterior part of the nucleus as a collar like microtubule structure (Toshimori and Ito, 2003). One major component is β-tubulin, which was co-stained with Sun4 to analyze the relation between Sun4 and the manchette. As shown in Fig. 1H, Sun4 is present only at regions where the cytoplasmic microtubules contact the NE, indicating that it is a component of the nucleocytoplasmic junction (see below).

Since the Sun4 distribution was strikingly reminiscent of Sun3 localization (Göb et al., 2010), we next asked, whether Sun4 distribution is actually identical to that of Sun3. Co-staining of Sun4 and Sun3, or Sun4 and Nesprin1, the LINC complex partner of Sun3 (Göb et al., 2010), revealed that the Sun4 signal indeed completely overlaps with Sun3 staining (Fig. 1I) and, consistently, also with Nesprin1 (Fig. 1J). Hence, all three proteins are present in the same region, i.e. the posterior lateral regions adjacent to the NE-associated microtubules. This points to a cooperative function of Sun3, Sun4 and Nesprin1 in connecting the microtubule manchette to the NE, supposed to be required for transferring cytoplasmic forces to shape the sperm nuclei (Göb et al., 2010; Kierszenbaum et al., 2011).

### Disruption of the Sun4 gene leads to male infertility

**Fig. 2.**
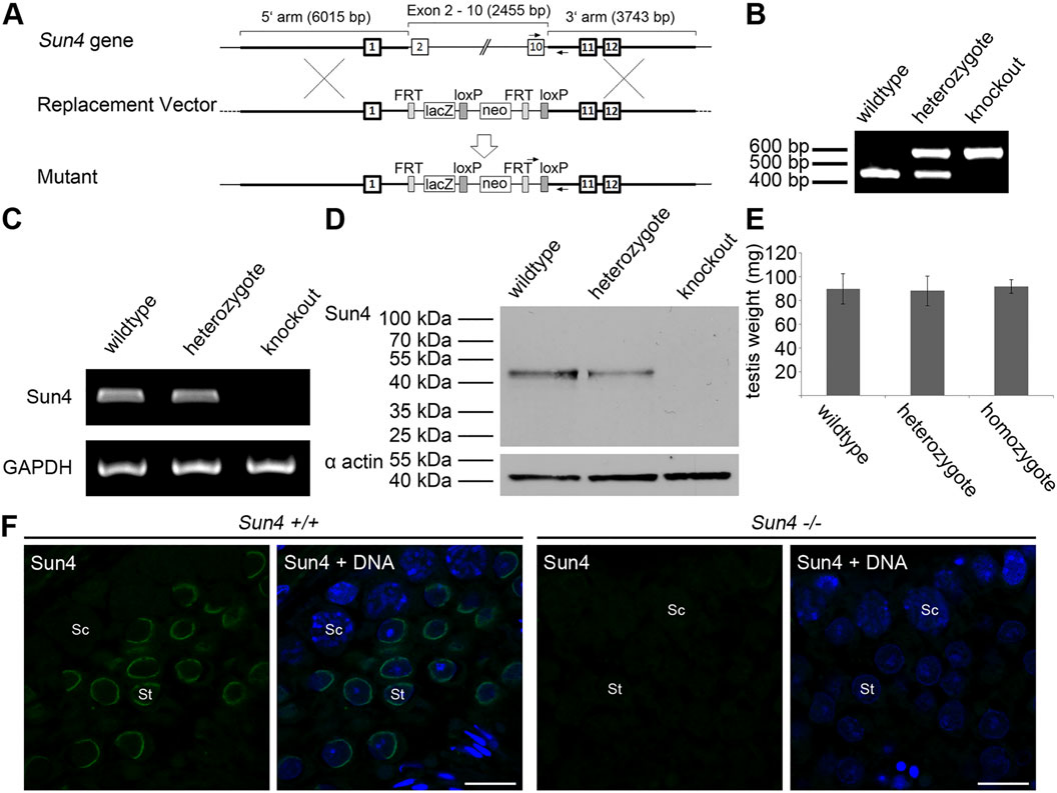
**Generation and characterization of Sun4-deficient mice.** (A) Structure of the *Sun4* gene, the replacement vector, which lacks exons two to ten, and the targeted mutant allele. Primer sites for genotyping are indicated by arrows. (B) Genotyping by PCR using the indicated primers for the wild-type and the mutant alleles (Table S1). (C) RT-PCR and (D) immunoblot analyses showing presence of Sun4 in wild-type and heterozygous, but Sun4 absence in homozygous mutant mice. (E) Comparisons of testis weight of wild-type (*n*=14), heterozygous (*n*=16) and homozygous (*n*=12) mutant mice show no difference. Error bars represent standard deviations of the mean values. (F) Immunohistochemical analyses on testis tissues demonstrating normal Sun4 expression in the wild-type, but its absence in *Sun4^−/−^* mice. Sc, spermatocyte; St, spermatid; Scale bars: 10 µm.

To investigate the role of Sun4 in nucleocytoskeletal linkage and sperm head formation, we next analyzed the consequence of Sun4 deficiency on sperm development and differentiation using a Sun4-deficient mouse line created by the KOMP Repository (www.komp.org; see Materials and Methods). Deletion of exons 2 to 10 within the *Sun4* (*Spag4*) gene leads to the loss of the coding regions for nearly all functional domains of Sun4, including the transmembrane domain, the coiled-coil region and the SUN domain (Fig. 2A). Correct gene targeting was verified by genotyping via PCR using specific 5′-oligonucleotides for either the wild-type or the knockout sequence and a common 3′-oligonucleotide to co-detect the wild-type and the knockout alleles (Fig. 2B). Accordingly, we could amplify a single fragment of 446 bp in the wild type or 593 bp in the knockout, or both in the heterozygote situation. *Sun4* gene disruption was confirmed on mRNA and protein level by performing RT-PCR or western blot analysis, respectively (Fig. 2C,D). Immunofluorescence microscopy on testis paraffin sections further evidenced absence of Sun4 in the knockout as Sun4-specific staining could not be detected in the *Sun4^−/−^* tissues (Fig. 2F).

Mating heterozygous males and females produced offspring with normal litter size and the mutated locus in Mendelian ratio, demonstrating that heterozygosity does not affect fertility and that Sun4 is not essential for normal embryonic development. Furthermore, *Sun4^−/−^* mice develop no primary visible phenotype regarding size and behavior. Repeated mating attempts of *Sun4^+/+^* females with *Sun4^−/−^* males did not produce offspring, implying that *Sun4^−/−^* males are infertile. However, mating of *Sun4^+/−^* males with *Sun4^−/−^* females had no overt negative effect on litter size, which is consistent with the notion that Sun4 is testis-specific.

### Sun4 deficiency severely interferes with sperm head formation

**Fig. 3.**
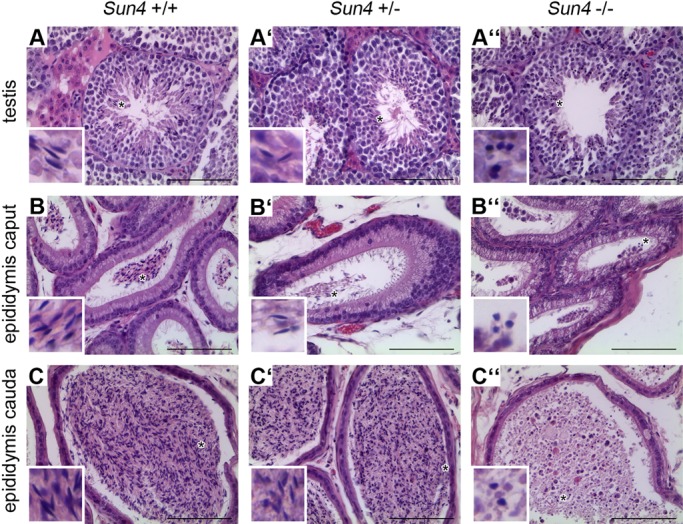
**Spermiogenesis is disturbed in Sun4-deficient mice.** Histological analysis of testis and epididymis tissues from *Sun4^+/+^* (A-C), *Sun4^+/−^* (A′-C′) and *Sun4^−/−^* (A″-C″) mice (15 weeks) stained with hematoxylin and eosin. While testes of *Sun4^+/+^*, *Sun4^+/−^* mice contain all typical spermatogenic stages (A,A′), elongated spermatids are absent in *Sun4^−/−^* tubules; instead, numerous abnormal spermatids with round nuclei appear (A″). Accordingly, *Sun4^−/−^* epididymis lack regular spermatozoa, but contain numerous aberrant germ cells with round nuclei in the lumen (B″,C″). Insets represent higher magnifications of the regions marked with asterisks. Scale bars: 50 µm.

Since *Sun4^−/−^* males were infertile, we next analyzed spermiogenesis in the Sun4-deficient background in more detail. Interestingly, as described for other genetic mouse models for male infertility caused by spermiogenic defects (Yan, 2009), Sun4 wild-type, heterozygous and knockout littermates did not reveal any overt difference regarding testis size and weight (Fig. 2E). To determine, whether Sun4 disruption actually impacts sperm development or differentiation, we performed histological analysis of testes and epididymis of adult *Sun4^+/+^*, *Sun4^+/−^* and *Sun4^−/−^* mice. As one would expect for a protein with postmeiosis-specific function, we could not find any overt differences between the three different genotypes concerning the somatic cells, spermatogonia and spermatocytes (Fig. 3A-A″). However, while early round spermatids also appeared quite normal in *Sun4^−/−^* males, we did not observe normal appearing elongated spermatids in the mutant testes. Instead, beginning with stage eight to nine of the spermatogenic cycle (see Russell et al., 1990) *Sun4^−/−^* tubules contained a huge number of aberrant roundish spermatids, indicating that sperm elongation and shaping is significantly disrupted (Fig. 3A″). Although in the absence of Sun4 sperm formation is severely affected, the aberrant shaped spermatozoa are not resorbed in the testes, but are still delivered to the epididymis (Fig. 3B″,C″).

**Fig. 4.**
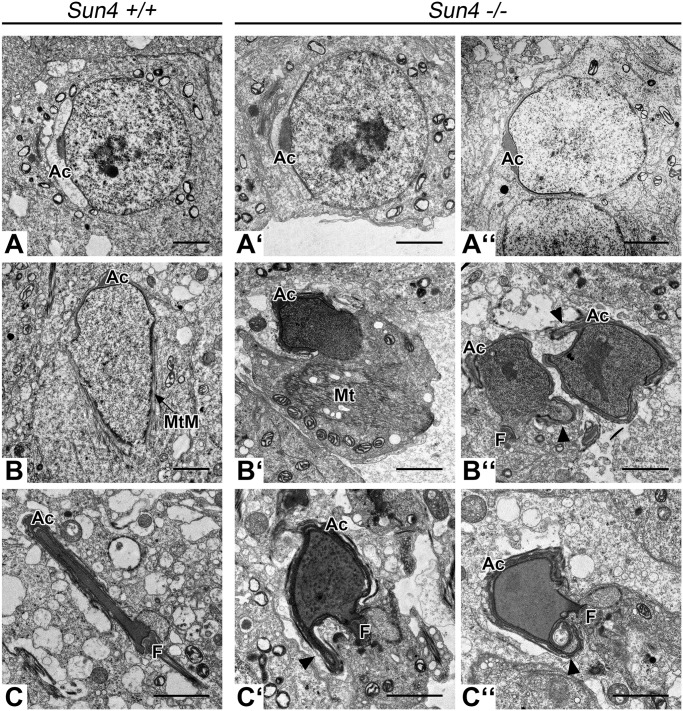
**Formation of the sperm head is severely impaired in *Sun4^−/−^* elongating spermatids.** Electron microscopic images of *Sun4^+/+^* (A-C) and *Sun4^−/−^* (A′-C″) spermatids. While early *Sun4^−/−^* spermatids appear to be normal (A′,A″), later *Sun4^−/−^* spermatid stages show distinctive malformations. Arrowheads indicate nuclear lobulations. Ac, Acrosome; F, Flagellar structures; MtM, microtubule manchette; Mt, microtubule structure. Scale bars: 2 µm.

To analyze the defects in more detail, we performed electron microscopy on ultrathin sections of testis and epididymis tissue from wild-type and knockout animals. Consistent with the histological analysis the premeiotic and meiotic stages of Sun4-deficient mice were phenotypically normal (not shown). Likewise, in early round spermatids we could not detect any morphological abnormalities, as nuclei appeared still round with the acrosome being properly attached at and expanding over the anterior NE (Fig. 4A,A″). However, instead of the later stages in sperm differentiation the *Sun4^−/−^* tubules contained a huge number of aberrant spermatids showing quite bizarre but coherent phenotypes (Fig. 4B′,B″). While differentiating wild-type spermatids develop typical flattened and elongated nuclei, the designated sperm heads of corresponding *Sun4^−/−^* stages were not elongated, but rather appeared completely misshaped (Fig. 4C-C″). Moreover, in most of the differentiating spermatids absence of Sun4 further caused profound nuclear membrane evaginations. Chromatin compaction, however, seems not to be affected as the mutant spermatids show visually normal appearing condensed chromatin. Notably, in most *Sun4^−/−^* spermatids the acrosome still covered the anterior side of the nuclei thereby aligning with the deformed NE, indicating that there is no general defect in acrosomal attachment. Likewise, formation and posterior positioning of the flagellum in *Sun4^−/−^* spermatids appeared to be wild-type-like (Fig. 4C-C″). Closer inspection of the nucleocytoplasmic junction revealed that the manchette, which in the wild type appears as a bundle-like structure connected to the posterior NE (Fig. 4B), was severely disorganized or even completely missing in the *Sun4^−/−^* spermatids (Fig. 4B′).

**Fig. 5.**
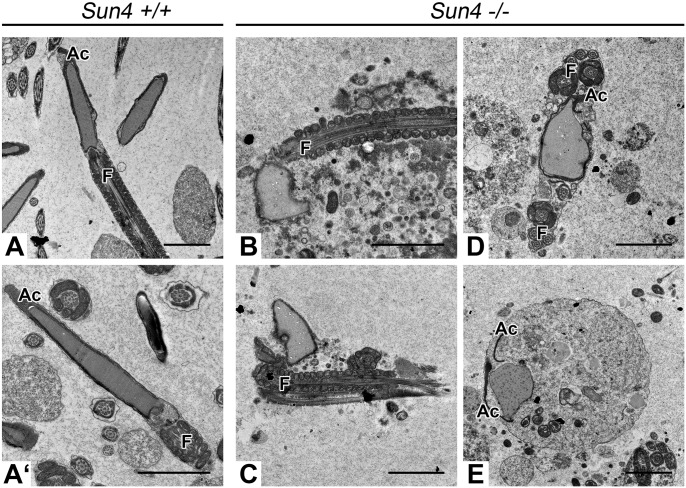
**Absence of Sun4 leads to a globozoospermia-like phenotype.** Representative electron micrographs of wild-type (A,A′) and *Sun4^−/−^* (B-E) spermatozoa within the epididymis. Compared to the wild-type, Sun4-deficient spermatozoa are characterized by malformed roundish nuclei. *Sun4^−/−^* spermatozoa occasionally are encircled by their tail (D) or sometimes are found in large constrictions, indicating cell degeneration (E). Ac, Acrosome; F, Flagellum. Scale bars: 2 µm.

EM-analysis of the epididymis confirmed the severe defects in sperm head formation as all spermatozoa in the *Sun4^−/−^* epididymal tubules were gravely misshaped (Fig. 5B-E). Additionally, during final maturation of Sun4-deficient spermatozoa, the flagella in several cases had tendency to coil around the nuclei and, occasionally, acrosomes were found partially detached from the NE (Fig. 5B-E), thus resembling a globozoospermia-like phenotype (Dam et al., 2011).

### Sun4 deficiency impairs the localization of other LINC components and associated structures

**Fig. 6.**
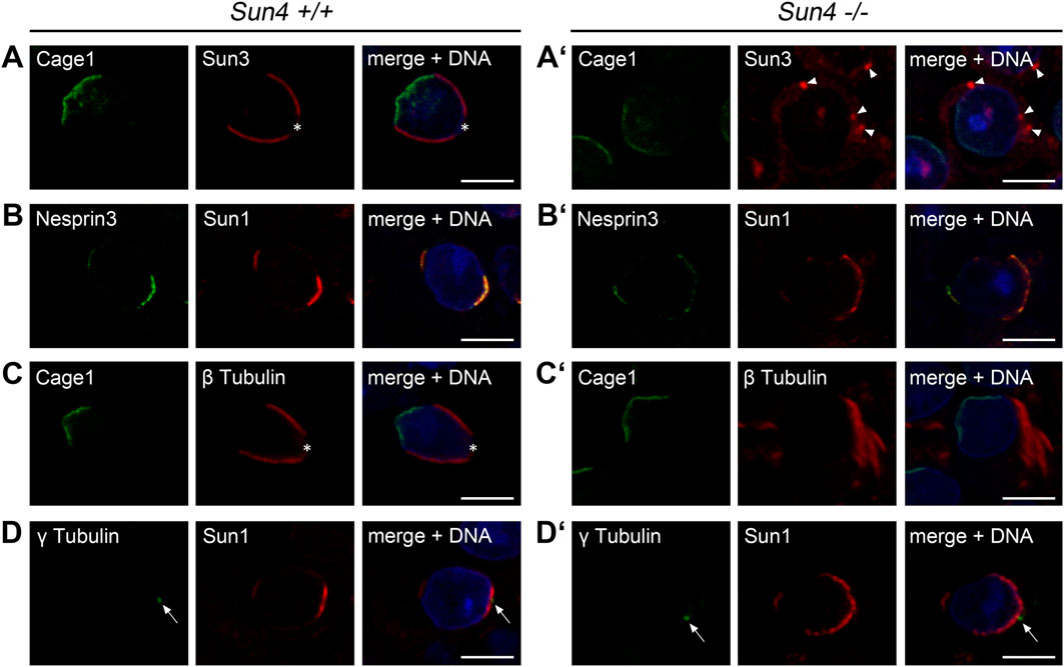
**Sun4 deficiency affects localization of other LINC components and NE**-**associated structures.** Testes paraffin sections of *Sun4^+/+^* and *Sun4^−/−^* littermates were co-stained for Cage1 and Sun3 (A,A′), Nesprin3 and Sun1 (B,B′), Cage1 and β-tubulin (C,C′), or γ-tubulin and Sun1 (D,D′). Overlays are depicted on the right side; DNA is stained in blue. Asterisks denote the implantation fossa, arrows indicate the basal body. Sun3 aggregations typically found within *Sun4^−/−^* spermatids are denoted by arrowheads. Scale bars: 5 µm.

To investigate the actual impact of Sun4 on organization of the spermatid NE and its associated structures, we next analyzed the localization of vital components of the spermatid nucleocytoplasmic junction. Since both Sun4 and Sun3 revealed virtual identical localization, we asked, whether absence of Sun4 affects Sun3 localization. Indeed, while in wild-type spermatids Sun3 showed its typical distribution within the posterior NE, in *Sun4^−/−^* spermatids Sun3 was seriously mislocalized: it could hardly be found at the NE, but rather appeared relocated to the cytoplasm with the tendency to accumulate in cytoplasmic aggregations (Fig. 6A,A′). Also, the interacting partner of Sun3, Nesprin1, appears to be affected as it could no longer be detected at the posterior NE of *Sun4^−/−^* spermatids (not shown). This clearly demonstrates that correct NE-localization of Sun3 and probably of Nesprin1 as well depends on the presence of Sun4.

Next we investigated whether Sun4 depletion has also consequences for localization of Sun1/Nesprin3 LINC complexes. In the wild type, during early spermiogenesis Sun1/Nesprin3 localizes at the posterior NE, excluding the implantation fossa, and at the anterior, acrosomal side of the sperm head. As elongation progresses, Sun1/Nesprin3 finally disappears from the posterior region and is only detected at the acrosomal pole of the sperm head (Göb et al., 2010). In the absence of Sun4, Sun1/Nesprin3 retained a dual localization, which in principle resembled the wild-type-like distribution. More detailed inspection, however, revealed that Sun4 deficiency yet has a mild but significant effect, as compared to the wild-type situation the posterior Sun1/Nesprin3 signals were found extending to more apical regions (Fig. 6B,B′). This suggests that Sun4 is required for accurate localization of the posterior Sun1/Nesprin3 LINC complexes as well.

**Fig. 7.**
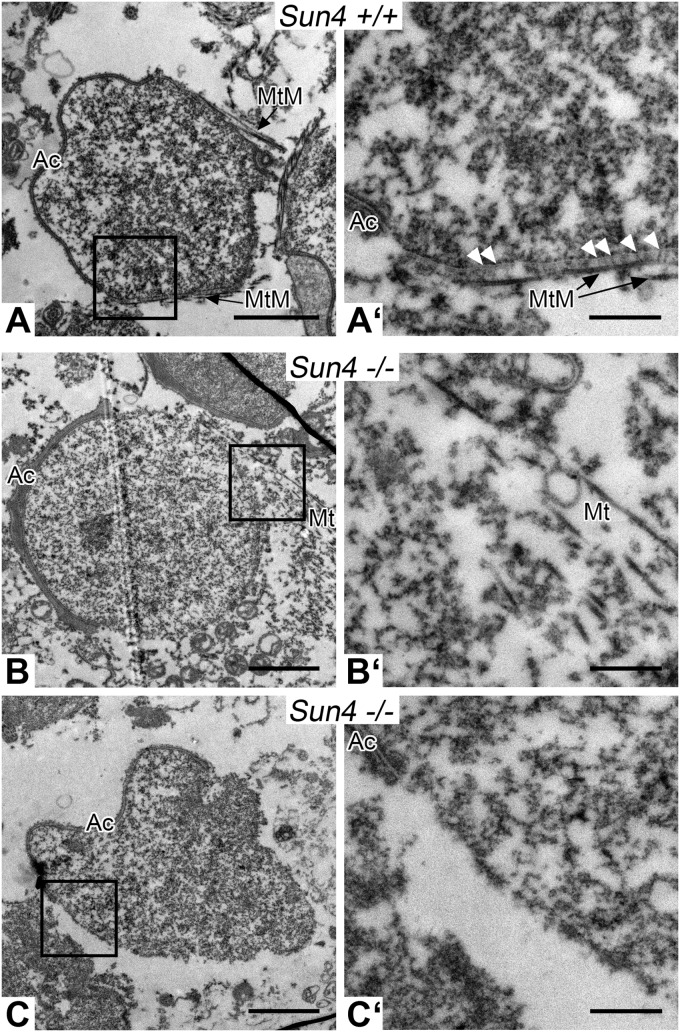
**Sun4-deficient mice lack the linkers between the NE and the microtubule manchette.** Electron microscopic images of *Sun4^+/+^* (A,A′) and *Sun4^−/−^* (B-C′) spermatids. (A,A′) Wild-type spermatids show rod-like filaments (arrowheads) between the NE and the closely associated microtubule manchette (MtM). In Sun4-deficient spermatids these structures are missing and the microtubules (Mt) appear loosely assembled and disorganized (B,B′) or, in most cases, can't even be found in the proximity of the nucleus (C,C′). Ac, Acrosome. Scale bars: 2 µm (A-C) and 500 nm (A′-C′).

Because posterior LINC complexes were proposed to be involved in connecting the manchette to the NE (Göb et al., 2010; Kierszenbaum et al., 2011), we then asked whether Sun4 deficiency impacts anchorage and/or formation of the manchette. As expected, in wild-type spermatids antibodies against β-tubulin nicely stained the manchette, which is seen closely associated with the posterior NE (Fig. 6C). By contrast, *Sun4^−/−^* spermatids failed to organize a typical manchette. Here, β-tubulin was still able to polymerize, but assembled to peculiarly disorganized filamentous arrays, which in most cases were formed in some distance to the NE (Figs 6C′ and 4B′). This was confirmed by detailed ultrastructural analysis of the nucleocytoplasmic junction (Fig. 7). Consistent with a previous study by Russell et al. (1991), using the nearly identical protocol in wild-type preparations we could identify numerous thin filaments arising from the nuclear surface and interconnecting the NE with the microtubule manchette (Fig. 7A,A′). Such filaments could be found only in the wild-type, but were completely missing in the *Sun4^−/−^* spermatids, which also show a disorganized manchette that is typically dissociated from the NE (Fig. 7B,B′ and C,C′). This clearly demonstrates that Sun4 is not only required for anchorage of the manchette, but is also crucial for its basic organization.

Since *Drosophila* Spag4, the presumed fly ortholog of mammalian Sun4, appears to be critical for NE attachment and proper positioning of the centriole/basal body during sperm development (Kracklauer et al., 2010), we next analyzed whether Sun4 deficiency has consequences for anchorage and/or positioning of the spermatid centriole/basal body in mammals as well. While in Spag4-deficient flies the basal body dissociates from the NE, thereby often shifting to one side of the nucleus (Kracklauer et al., 2010), γ-tubulin staining in *Sun4^−/−^* spermatids demonstrates that the centriole/basal body position in relation to the posterior NE remained the same as in the wild type (Fig. 6D,D′). This together with the fact that the anterior acrosome is still in place suggests that the general polarity is preserved in *Sun4^−/−^* spermatids.

Our results so far demonstrate that Sun4 has a significant influence on the localization of other posterior LINC components. Furthermore, Sun4 deficiency severely impacts assembly and anchorage of the manchette. However, Sun4 appears neither involved in centriole/basal body attachment to the NE, nor in setting up and maintaining a more general spermatid-specific cell or nuclear polarity.

### Sun4 has the intrinsic property to interact with Sun3, Nesprin1 and itself

**Fig. 8.**
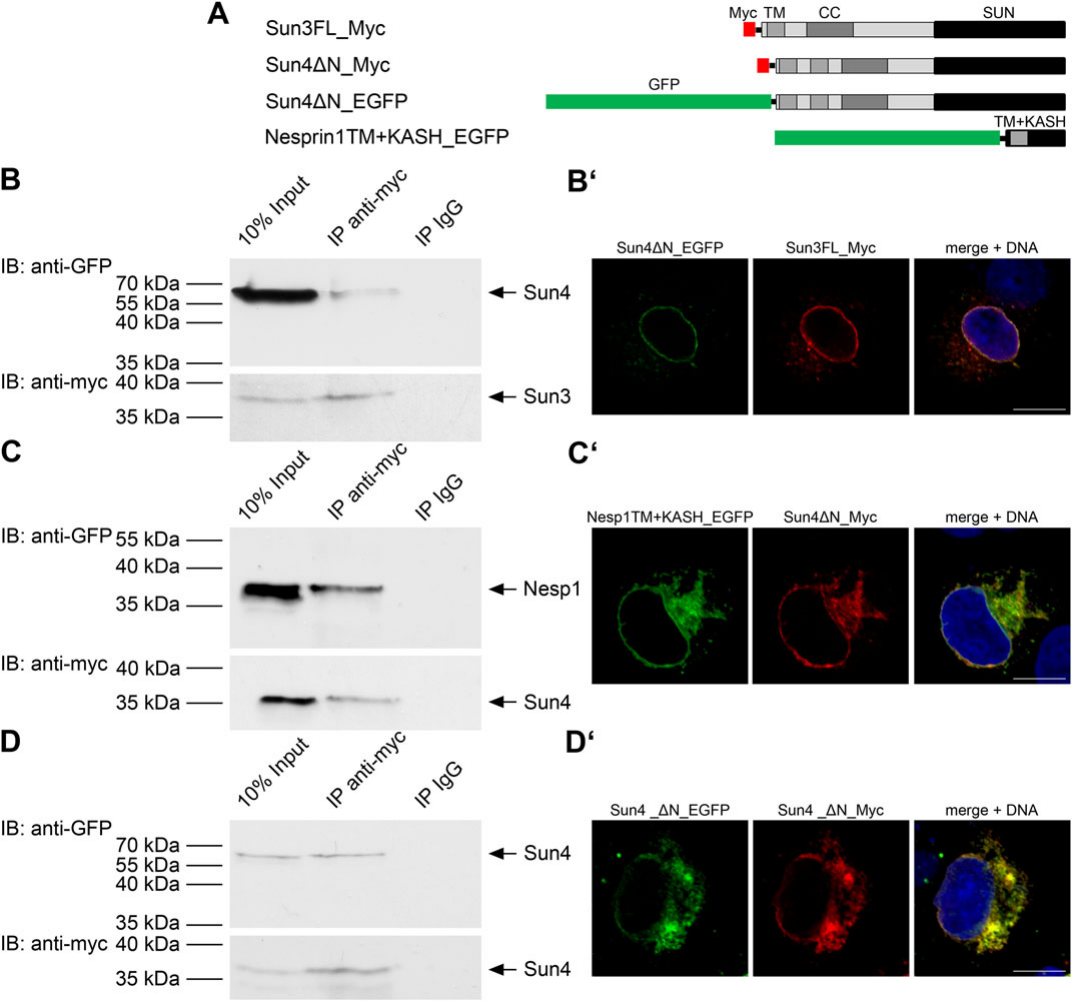
**Sun4 is capable of binding to Sun3, Nesprin1 and to itself.** (A) Constructs used for transfection/co-immunoprecipitation experiments. COS-7 cells were co-transfected with EGFP-Sun4 and Myc-Sun3 (B), EGFP-Nesprin1 and Myc-Sun4 (C), or EGFP-Sun4 and Myc-Sun4 (D). Cells lysed in RIPA buffer were co-immunoprecipitated with anti-myc mAbs and detected with either anti-EGFP or anti-myc antibodies. For control, unspecific rabbit IgG antibodies were used. (B′-D′) Confocal images of corresponding transfections visualized by immunofluorescence. Scale bars: 10 µm.

Our results above support the idea that Sun4 may interact with Sun3 and/or Nesprin1 to fulfill a cooperative function during sperm head formation. To study this possibility we performed co-transfection/co-immunoprecipitation experiments according to previous approaches to test SUN-KASH interactions (Stewart-Hutchinson et al., 2008; Göb et al., 2010). Different combinations of Myc- and EGFP-tagged constructs of Sun3, Sun4 and Nesprin1 (Fig. 8A) were co-expressed in COS7 cells and immunoprecipitated with either anti-Myc or anti-GFP antibodies. In this assay we could co-immunoprecipitate EGFP-tagged Sun4 protein with Myc-tagged Sun3 as bait (Fig. 8B). Furthermore, Myc-tagged Sun4 was also able to bring down the EGFP-tagged KASH domain of Nesprin1 (Fig. 8C). This demonstrates that ectopically expressed Sun4 is capable to interact with Sun3 and the KASH domain of Nesprin1. Since SUN proteins were supposed to form homo- or, contingently, heterotrimeric assemblies, which interact with corresponding KASH trimers (Sosa et al., 2012), we further analyzed whether Sun4 can also bind to itself to form oligomeric LINC assemblies. Indeed, EGFP-tagged Sun4 could be co-immunoprecipitated with Myc-tagged Sun4, demonstrating that Sun4 has the property to interact with itself (Fig. 8D). To test whether Sun3-Sun4 heteromeric interactions occur in spermatids we performed structured illumination microscopy (SIM) and compared their distribution on surface views of the posterior NE. Both proteins showed only occasional overlap suggesting that Sun3 and Sun4 preferentially form homotrimers, but to some extent they also co-localized, indicating that they may interact with each other and form heterotrimeric assemblies or intermediates as well (Fig. S2). In any case, our results demonstrated that Sun4 has the capacity to interact with Sun3 and Nesprin1 and, furthermore, with itself.

## DISCUSSION

Nuclear remodeling and elongation are prominent characteristics of sperm differentiation, pivotal to generate fertilization competent spermatozoa. Failures in underlying processes could provoke misshaping of the sperm head, which often leads to male infertility (Yan, 2009). Despite the vital role of nuclear reshaping, very little is known about how sperm nuclei are reorganized and shaped to realize their typical, species-specific elongated form. Previous studies suggested that the nucleocytoplasmic intersection could hold a key function in nuclear reorganization and shaping during sperm head formation. In particular, the NE could serve as a platform to transfer cytoplasmic forces to shape the spermatid nuclei. In this respect, LINC complexes are of particular interest as they may act as the primary connectors to the NE (Russell et al., 1991; Kierszenbaum and Tres, 2004; Göb et al., 2010; Kracklauer et al., 2010, 2013; Kierszenbaum et al., 2011). Consistently, distinct spermiogenesis-specific LINC components have been identified, which are located at opposite poles of the developing sperm head, appropriate to mediate opposing forces to the elongating nucleus: Sun1η/Nesprin3 and Sun5 localize to the anterior acrosomal side, whereas Sun3/Nesprin1 and, as shown here, Sun4 are found at the posterior NE (Göb et al., 2010; Kracklauer et al., 2013). The actual function of these components, however, remained unknown. We in our present study focused on Sun4 and analyzed its specific properties, behavior and function to gain insights into the role of LINC complexes during sperm differentiation.

### Sun4 is part of a posterior LINC complex, required for correct targeting of Sun3/Nesprin1

Consistent with previous results (Shao et al., 1999), we could detect Sun4 only in the testis and found it solely expressed in the haploid spermatids. Its expression pattern resembles that of Sun3 and closely correlates with sperm elongation and differentiation (Göb et al., 2010). Like described for Sun3 and Nesprin1 (Göb et al., 2010), Sun4 localizes to the posterior NE of round and elongating spermatids, which at least in part agrees with the notion that Spag4 (Sun4) can be found at the spermatid manchette (Shao et al., 1999). In contrast to the previous report, however, despite using three different antibodies directed against two different epitopes of Sun4, we could not trace Sun4 within the flagellum (Fig. S1). Thus, our results demonstrate that Sun4 is actually not, as previously stated, a component of the ODF of the axoneme, rather it is solely part of the posterior nucleocytoplasmic junction, located at sites where the microtubule manchette contacts the NE (Fig. 1E-J). This is remarkable, because Sun4 was found to interact with Odf1, a main ODF component within the flagellum (Petersen et al., 1999; Shao et al., 1999). Our finding does not argue against Sun4 interaction with Odf1, but locally confines such an interaction to the posterior NE, which is consistent with the recently reported role of Odf1 in sperm head-to-tail coupling (Yang et al., 2014).

Because of the congruency between Sun4 and Sun3/Nesprin1 distribution it appeared conceivable that they interact with each other to cooperate at the NE-manchette-intersection. In line with this, we demonstrated that Sun4 has the capacity to bind both Sun3 and Nesprin1. High resolution SIM analysis provided evidence that Sun4 and Sun3 preferentially form homotrimers. However, co-localization could be observed to some extent as well, indicating that Sun4 and Sun3 may also form heterotrimeric assemblies or intermediates (Fig. S2). Remarkably, absence of Sun4 leads to mislocalization of Sun3/Nesprin1, i.e. they disappear from the NE and relocate to the cytoplasm, suggesting that interaction with Sun4 is required for correct targeting of Sun3/Nesprin1. According to bioinformatic predictions, the putative nucleoplasmic domain of Sun3 consists of only seven amino acids, which presumably is too short to efficiently anchor Sun3 to nuclear structures (Göb et al., 2010). This could well explain previous findings that, when ectopically expressed in somatic cells Sun3 is retained in the ER, whereas in its natural environment Sun3 is recruited to the posterior NE, likely via binding to Sun4 (Crisp et al., 2006; Göb et al., 2010). Structural analyses of LINC complexes revealed a trimeric organization of SUN proteins, which bind three KASH peptides (Sosa et al., 2012). Taking into account that Sun4 has the competence to bind Sun3, Nesprin1 and itself, we suggest that Sun4 and Sun3 may form homo- or heterotrimers within the INM, which in the PNS interact with ONM bridging Nesprin1 to connect to the cytoplasmic microtubule arrays. Such LINC complexes could well represent the rod-like structures described by Russell et al. (1991), which connect the innermost microtubules of the manchette with the outer leaflet of the NE. Consistently, in our present study we found that such rod-like filaments are completely missing in *Sun4^−/−^* spermatids (Fig. 7).

### Loss of Sun4 impacts NE integrity but not general nuclear polarity

We could here demonstrate that Sun4 is crucial for correct localization of Sun3 and Nesprin1. Moreover, *Sun4^−/−^* spermatids show also significant changes in the distribution of other NE components, as Sun1 and Nesprin3 scatter from the posterior to more anterior regions of the NE (Fig. 6B′). Noteworthy, proteins within the spermatid NE are commonly not homogeneously distributed, they rather occupy well confined territories apparently related to their specific function (Göb et al., 2010; Frohnert et al., 2011; this study). In the absence of Sun4 this strictly regulated territoriality appears impaired, indicating that the posterior LINC complexes consisting of Sun3 and/or Sun4 and Nesprin1 may be involved in regulating NE partitioning or at least in providing some kind of barrier for Sun1/Nesprin3 LINC complex distribution. Sun4 deficiency not only affects distribution of NE proteins, but overtly interferes with general NE integrity: loss of Sun4 induces nuclear membrane protrusions and severe nuclear deformations (Fig. 4), which could be result of a destabilized membrane system and/or consequence of a disturbed cytoplasmic force transmission to the NE.

Interestingly, our immunofluorescence and EM data show that with Sun4 absence both the acrosome and the basal body still reside at their designated locations, which are the anterior pole and the posterior implantation fossa, respectively (Figs 4-6). This suggests that, though Sun4 deficiency impacts NE integrity, Sun4 is factually not required to establish and maintain general polarity of the sperm head, at least in the mouse.

### Sun4 is essential for correct assembly of the cytoplasmic microtubule manchette, but not for centriole attachment

Our results demonstrate that Sun4 deficiency severely impairs the assembly and organization of the cytoplasmic microtubule manchette (Figs 4, 6 and 7). A typical wild-type manchette is characterized by highly organized microtubule bundles that form a characteristic perinuclear rim surrounding the posterior NE (Kierszenbaum et al., 2011). In *Sun4^−/−^* spermatids microtubules were always found seriously disorganized. Tubulin was found to polymerize in aberrant microtubule arrays, which in most cases were completely dissociated from the NE. This clearly supports a direct functional interaction between the NE and the manchette (Göb et al., 2010; Kierszenbaum et al., 2011; Kracklauer et al., 2013). Consistently, in *Sun4^−/−^* spermatids the observed manchette disorganization closely correlates with severe nuclear deformations and impairment of nuclear elongation. Beyond this, our results provide evidence that LINC complexes not only interact with the cytoskeleton, but are vitally involved in organizing the cytoplasmic microtubule system, at least during sperm differentiation.

A previous study reported that *Drosophila* Spag4 is required for connecting the centriole/basal body to the NE in spermatids (Kracklauer et al., 2010). This is consistent with the general view that LINC complexes are used to anchor the microtubule organizing centers to the NE (Razafsky and Hodzic, 2009; Graumann et al., 2010; Starr and Fridolfsson, 2010; Yamamoto, 2014). In contrast to Spag4 in the fly, Sun4 in mammals seems to be dispensable for centriole-nuclear attachment and positioning of the basal body as in our study we could find centrioles always located near the implantation fossa, even in the absence of Sun4. Compared to *Drosophila* the mammalian NE is distinguished by significant increased complexity. While *Drosophila* expresses Spag4 as the only SUN domain protein during spermiogenesis, mammalian spermatids contain at least four different SUN domain proteins: Sun1/Sun1η and the testis-specific Sun3, Sun4 and Sun5 (Shao et al., 1999; Göb et al., 2010; Frohnert et al., 2011). This diversity allows for specialization of individual LINC complexes, but may also account for partial redundancy as described for Sun1 and Sun2 in somatic and meiotic cells (Ding et al., 2007; Lei et al., 2009; Link et al., 2014). Accordingly, Sun1, which in Sun4-deficient spermatids remains at the posterior NE, may compensate for Sun4 function in attaching the basal body to the NE. This is an interesting aspect worth clarifying in the future.

### Sun4 is an essential determinant for sperm head formation and fertility

A most striking consequence of Sun4 deficiency is impairment of elongation and shaping of the sperm head, leading to substantially deformed non-functional spermatozoa (Fig. 5). While early round *Sun4^−/−^* spermatids appear normal, the nuclei of later stages were seriously misshaped, showing grave deformations and lobulations of the NE. Consistent with the idea of an active role of LINC complexes in determining nuclear shape (Olins et al., 2009; Göb et al., 2010; Oda and Fukuda, 2011; Zhou et al., 2012; Kracklauer et al., 2013), here we provide direct evidence that during sperm formation LINC components are crucial in directing nuclear shaping, most likely by mediating force transmission to the NE. Interestingly, more than two decades ago, Russell and colleagues reported on rod-like elements linking the innermost microtubules of the manchette to the outer surface of the NE and across the PNS to the inner leaflet of the NE (Russell et al., 1991). From our study it appears likely that such filaments are comprised of LINC complexes formed by Sun4 and/or Sun3 and Nesprin1, which could connect to a cytoplasmic motor system (Russell et al., 1991; Dadoune, 2003; Toshimori and Ito, 2003; Göb et al., 2010; Kracklauer et al., 2013; Cartwright and Karakesisoglou, 2014). Accordingly, in Sun4-deficient spermatids the connection between the NE and the cytoplasmic microtubule manchette is disrupted, as demonstrated by the missing rod-like elements in the ultrastructural micrographs (Fig. 7). They further show serious manchette misarrangement and severe malformation of the sperm head (Fig. 4). As a consequence, Sun4-deficient males produce spermatozoa with a globozoospermia-like phenotype (Fig. 5), which are delivered to the epididymis, but are nonfunctional as evident from the infertility of male *Sun4^−/−^* mice. Similar globozoospermia-like phenotypes were reported for other mouse lines, including *Azh*, *Gopc* and *Hrb* mutant mice (Mochida et al., 1999; Suzuki-Toyota et al., 2004; Juneja and van Deursen, 2005; Suzuki-Toyota et al., 2007). While Sun4 is a posterior NE component, the responsible proteins in these cases were found to be cytoplasmic, likely involved in acrosome formation or head to tail coupling (for review see Kierszenbaum et al., 2011). Why the functional inactivation of such different proteins leads to similar phenotypes is not known and remains an interesting task for the future.

Taken together, our data presented here suggest that Sun4, which is exclusively found in posterior spermatid regions that are decorated by cytoplasmic microtubules, is an essential partner of Sun3 and Nesprin1. Sun4 containing LINC complexes seem to determine and coordinate the localization of other NE components and, surprisingly, the correct assembly of the microtubule manchette. In that, Sun4 is an essential determinant of sperm head formation and disruption of the Sun4 gene provokes serious malformation of the developing spermatid. This involves altered localization of NE components, disorganization of the microtubule manchette, NE lobulation and severe deformation of the spermatid nuclei. These failures finally manifest in a globozoospermia-like phenotype and lead to male infertility.

## MATERIALS AND METHODS

### Ethics statement

Animal care and experiments were conducted in accordance with the guidelines provided by the German Animal Welfare Act (German Ministry of Agriculture, Health and Economic Cooperation). Animal housing and breeding was approved by the regulatory agency of the city of Würzburg (Reference FB VVL 568/300-1870/13; according to §11/1 No. 1 of the German Animal Welfare Act). All aspects of mouse work were carried out following strict guidelines to ensure careful, consistent and ethical handling of mice.

### Animals and tissue preparation

Tissue samples were obtained from wild-type, heterozygous and knockout littermates derived from the *Spag4^tm1(KOMP)Mbp^* mouse strain. This strain was generated by the KOMP Repository (www.komp.org; Spag4 targeting project: 24921, design ID: 234716) from ES cells created by the CSD consortium with funding by the trans-NIH Knock-Out Mouse Project (KOMP) (Grant # 5U01HG004080). Tissues were excised from CO_2_-euthanized mice and processed for RNA isolation, protein analysis, histological or immunofluorescence studies.

### Genotyping of the transgenic Sun4 mice

Verification of gene targeting and genotyping of *Spag4^tm1(KOMP)Mbp^* offspring was done by PCR, using 300 ng of genomic DNA. Primers were selected as suggested by the KOMP Repository: 5′-oligonucleotide S4_CSD_vec5′ko and 3′-oligonucleotide SD_Spag4_SRI_3′ for the targeted allele, and the additional 5′-primer S4_Ex10_5′wt to co-detect the wild-type allele (Table S1), which amplify a 593 bp and/or a 446 bp fragment, respectively.

### cDNA cloning and generation of plasmid constructs

Total RNA was isolated from testicular suspensions of adult mice using TriFast™ (Peqlab Biotechnology) according to manufacturer's protocol. Reverse transcription was performed on 1 µg of total RNA with oligo(dT) primer and M-MLV reverse transcriptase (Promega). Based on the NCBI reference sequence NM_139151, a 5′-oligonucleotide upstream of the putative start codon (Sun4_5′) and a 3′-oligonucleotide downstream of the predicted stop codon (Sun4_3′) were used for PCR amplification of the full-length cDNA (Table S1). The product was cloned into the StrataClone Blunt PCR Cloning vector (pSC-B-amp/kan; Agilent Technologies) and verified by standard sequencing. For antibody production, a Sun4 fragment coding for amino acids 11-127 was amplified with primers S4_Nterm_5′_Nde and S4_Nterm_3′_Sal (Table S1) and cloned into pET-21a(+) expression vector (Novagen). To generate GFP- and Myc-tagged Sun4 constructs, the coding region corresponding to amino acids 135-444 was amplified with primers Sun4_ΔN_5′ and Sun4_SUNdom_3′ (Table S1) and cloned into either the pEGFP-C1 or the pCMV-Myc vector (Clontech Laboratories). Other constructs used in this study were described previously (Göb et al., 2010).

### Expression analysis by RT-PCR

The expression profile of *Sun4* was analyzed by RT-PCR on total RNA, isolated from selected tissues of adult mice or from testicular suspensions of pubertal mice (8-25 days). Reverse transcription was performed as described above. To monitor *Sun4* mRNA expression we used primer pair, Sun4_cc_5′; Sun4_cc_3′ (Table S1), flanking the putative coiled coil region of Sun4 for PCR amplification.

### Cell culture and transfection

COS-7 cells were grown at 37°C and 5% CO_2_ in DMEM (Invitrogen) supplemented with 10% FCS and 1% penicillin/streptomycin. Cells were transfected with the respective pEGFP- and pCMV Myc expression vectors using Effectene™ (Qiagen) according to manufacturer's instructions and further incubated overnight.

### Antibodies

To raise antibodies against the Sun4 N-terminal domain, we expressed a His-tagged Sun4 fusion construct coding for amino acids 11 to 127 of full length murine Sun4, purified the protein under denatured conditions on Ni-NTA agarose resin (Qiagen) and used it to immunize guinea pigs and rabbits (BioScience, Göttingen, Germany). Alternatively, a small peptide corresponding to amino acids 246-260 was synthesized (BioScience) and immunized to generate antibodies against a Sun4 perinuclear domain. Sera were affinity purified with HiTrap™ NHS-activated HP columns (GE Healthcare) coupled with the respective antigen according to the manufacturer's instructions. Other primary antibodies used in this study were: rabbit anti-Cage1, guinea pig anti-Sun3 and guinea pig anti-Sun1, rabbit anti-Nesprin3, rabbit anti-SYNE1, mouse anti-β-tubulin, rabbit anti-γ-tubulin, mouse anti-actin, mouse anti-GFP (B-2) and mouse anti-Myc. Details about sources and working dilutions of each of the primary antibodies are listed in Table S2. Corresponding secondary antibodies were purchased from Dianova (Hamburg, Germany).

### Co-immunoprecipitation

Co-immunoprecipitations were conducted as previously described (Göb et al., 2010). Selected Myc- and EGFP-fusion constructs were co-expressed in COS-7 cells, Myc-tagged proteins were immunoprecipitated with anti-myc antibodies (#06-549, Upstate/Millipore) and putative co-precipitating EGFP-fusion proteins were detected with anti-EGFP antibodies.

### SDS-PAGE and western blotting

Protein samples derived from co-immunoprecipitation experiments, mouse tissues or testicular cells were resuspended in 2× SDS sample buffer and heated to 95°C for several minutes. Proteins were separated on SDS-PAGE according to standard procedures and transferred to nitrocellulose membranes. After overnight blocking at 4°C in TBST (10 mM Tris/HCl, 150 mM NaCl, 0.1% Tween 20, pH 7.4) supplemented with 10% milk, membranes were incubated for one hour at room temperature with respective primary antibodies in blocking solution. After washing in TBST, membranes were incubated with appropriate peroxidase coupled secondary antibodies. Signals were detected using Western Lightning^®^ Plus-ECL Substrate (Perkin Elmer).

### Immunocytochemistry

Testes were fixed for three hours at room temperature in 1% PBS-buffered paraformaldehyde. After washing in PBS, tissues were dehydrated and embedded in paraffin as described previously (Alsheimer et al., 2005). Sections of 4 to 5 µm were placed on slides, dewaxed, rehydrated and antigen retrieval was performed (Alsheimer et al., 2005). Samples were permeabilized with 0.1% Triton X-100 for 10 min, washed in PBS and blocked for 1 h with PBT (0.15% BSA, 0.1% Tween 20 in PBS), incubated for one hour with respective primary antibodies, and after washing with PBS incubated with appropriate secondary antibodies for 20 min. To counterstain DNA, Hoechst 33258 (Serva) was added for additional 10 min.

### Microscopy and image analysis

Immunostainings were visualized using Leica TCS-SP2 AOBS confocal laser scanning microscope equipped with 63×/1.40 HCX PL APO oil-immersion objective (Leica Microsystems, Wetzlar, Germany). Confocal images shown are calculated maximum projections of sequences of three proximate single sections. SIM analysis was carried out on singularized spermatids from testis suspensions, which were fixed with 2% formaldehyde in PBS and spread on slides. After gentle drying, samples were further processed for immunolabeling as described above. SIM was conducted using Zeiss ELYRA S.1 equipped with Zeiss Plan-Apochromat 63×/1.4 oil DIC objective and run with Zeiss ZEN 2012 software package including Superresolution Structured Illumination plugin (Carl Zeiss Microscopy GmbH, Jena, Germany). Images were processed with ImageJ (National Institutes of Health, version 1.42q; http://rsbweb.nih.gov/ij) and Adobe Photoshop CS5 (Adobe Systems).

### Histology

Histological analysis was performed on 5 µm sections of paraffin-embedded testes tissues that were fixed overnight in 4% paraformaldehyde. After dewaxing and rehydration sections were stained with Hematoxylin and Eosin according to standard protocols. Images were taken using Olympus CX41 microscope equipped with Leica EC3 camera.

### Electron microscopy

Testes or epididymis tissue pieces were fixed for 45 min in 2.5% glutaraldehyde (2.5% glutaraldehyde, 50 mM KCl, 2.5 mM MgCl, 50 mM cacodylate; pH 7.2) and washed in cacodylate buffer (50 mM; pH 7.2). Following postfixation for 1 h at 4°C with 2% osmium tetroxide in 50 mM cacodylate, samples were washed several times in H_2_O and stained at 4°C overnight using 0.5% uranyl acetate. Tissues were dehydrated in an increasing ethanol series, incubated three times in propylene oxide for 30 min each and embedded in Epon. Ultrathin sections were double stained with uranyl acetate and lead citrate according to standard procedures and analyzed with JEM-2100 transmission electron microscope (Jeol, Eching, Germany).

For analysis of the connection between the NE and the microtubule manchette, testicular suspensions were treated as described by Russell et al. (1991). Samples were stained with 2% uranyl acetate overnight at 4°C and further processed as described above.
